# *FBN2* Silencing Recapitulates Hypoxic Conditions and Induces Elastic Fiber Impairment in Human Dermal Fibroblasts

**DOI:** 10.3390/ijms23031824

**Published:** 2022-02-05

**Authors:** Jérémy Boizot, Mélaine Minville-Walz, Dieter Peter Reinhardt, Marielle Bouschbacher, Pascal Sommer, Dominique Sigaudo-Roussel, Romain Debret

**Affiliations:** 1 CNRS UMR 5305, LBTI, 7 Passage du Vercors, CEDEX 7, 69367 Lyon, France; jeremyboizot@yahoo.fr (J.B.); pascal.sommer@univ-amu.fr (P.S.); dominique.sigaudo-roussel@ibcp.fr (D.S.-R.); 2 University of Lyon 1, UFR Biosciences, 7 Passage du Vercors, CEDEX 7, 69367 Lyon, France; 3 Urgo Research Innovation and Development, 42 Rue de Longvic, 21300 Chenôve, France; m_walz@yahoo.fr (M.M.-W.); m.bouschbacher@fr.urgo.com (M.B.); 4 Department of Anatomy and Cell Biology, Faculty of Medicine, McGill University, Montreal, QC H3A 0C7, Canada; dieter.reinhardt@mcgill.ca; 5 Faculty of Dentistry, McGill University, Montreal, QC H3A 0C7, Canada

**Keywords:** fibrillin-2, elastin, elastic fiber, hypoxia, skin wound

## Abstract

Most chronic wounds are characterized by varying degrees of hypoxia and low partial pressures of O_2_ that may favor the development of the wound and/or delay healing. However, most studies regarding extracellular matrix remodeling in wound healing are conducted under normoxic conditions. Here, we investigated the consequences of hypoxia on elastic network formation, both in a mouse model of pressure-induced hypoxic ulcer and in human primary fibroblasts cultured under hypoxic conditions. In vitro, hypoxia inhibited elastic fiber synthesis with a reduction in fibrillin-2 expression at the mRNA and protein levels. Lysyl oxidase maturation was reduced, concomitant with lower enzymatic activity. Fibrillin-2 and lysyl oxidase could interact directly, whereas the downregulation of fibrillin-2 was associated with deficient lysyl oxidase maturation. Elastic fibers were not synthesized in the hypoxic inflammatory tissues resulting from in vivo pressure-induced ulcer. Tropoelastin and fibrillin-2 were expressed sparsely in hypoxic tissues stained with carbonic anhydrase IX. Different hypoxic conditions in culture resulted in the arrest of elastic fiber synthesis. The present study demonstrated the involvement of FBN2 in regulating elastin deposition in adult skin models and described the specific impact of hypoxia on the elastin network without consequences on collagen and fibronectin networks.

## 1. Introduction

Chronic wounds are associated with vascular diseases, diabetes mellitus or aging, and present a major and yet unresolved clinical problem. They are characterized by the premature senescence of fibroblasts [1], uncontrolled inflammation and a remodeling process that does not reconstitute the initial properties of tissues [2]. Most chronic wounds are characterized by varying degrees of hypoxia and low partial pressures of O_2_ that may favor the development of the wound and/or delay healing. Hypoxia, often occurring in chronic wounds, has a leading role in extracellular matrix (ECM) remodeling; it has been shown to increase in venous leg ulcers and was related to the clinical severity of the wound [3,4,5,6]. Diabetic foot ulcers are a clinical outcome of different causes including peripheral hypoxia. However, little is known on the direct involvement of hypoxia-related extracellular matrix alteration in chronic wounds [7].

In skin, elastic fibers are synthesized by fibroblasts, and their functional assembly is often considered as a limiting step in the healing process [8,9]. The soluble precursor of elastin (ELN), tropoelastin, acquires its mechanical properties once deposited in an orderly manner on microfibrils, composed mainly of fibrillin-1 and -2 (FBN1, FBN2). Other proteins, such as microfibrillar associated protein (MAGP)-1, small fibulins and emilins, are also involved in this elastic fiber network [10,11]. Tropoelastin is cross-linked after the action of specific enzymes, the lysyl oxidases (LOXs), mainly LOX and LOXL1, which catalyze the oxidative deamination of lysine residues. Once ELN is cross-linked and associated with microfibrils, the resulting fibers are extremely resistant to environmental stress, tensile strength and compression [12]. LOX and LOXL1 are both involved in wound healing [13,14,15,16]. They belong to the same family and are processed by bone morphogenic protein 1 (BMP-1) and related proteases [17]. In contrast to fetal wound healing, in the case of adult injury or in chronic wounds, when elastic fibers are degraded, such tissue has a weak capacity to renew a functional elastic network [8]. Although the link between hypoxia that is present in injured tissue and elastogenesis has never been investigated, studies show that hypoxia affects the expression of tropoelastin and LOX, which can potentially disrupt fiber synthesis elastics in the healing process [18,19]. In addition, since in vitro studies are usually conducted under normoxia (21% pO_2_), the originality of our study is therefore based on the analysis of elastogenesis in an intermittent or extended hypoxic environment (1% pO_2_) imposed on the cells to mimic, at least in part, the alteration of tissue oxygenation conditions.

In order to investigate hypoxia-related mechanisms involved in skin lesion and healing that might hinder the synthesis of elastic tissues, we questioned whether hypoxia could modulate elastin neo-formation. To address this, we designed in vitro protocols to submit fibroblasts to several pattern of hypoxic stress and an in vivo protocol to submit the skin to hypoxia using the pressure-induced ulcer model [20]. We demonstrated that hypoxia affects elastic fibers’ deposition and FBN2, but not type I collagen deposition nor fibronectin. Our findings also show that FBN2 silencing under normoxia recapitulates the in vitro hypoxia condition with both reductions in elastic fiber deposition and LOX maturation. Finally, the association between FBN2 and ELN persisted in vivo under cutaneous tissue hypoxia challenge.

## 2. Results

### 2.1. Setting-Up In Vitro Hypoxic Conditions

**Figure 1:**
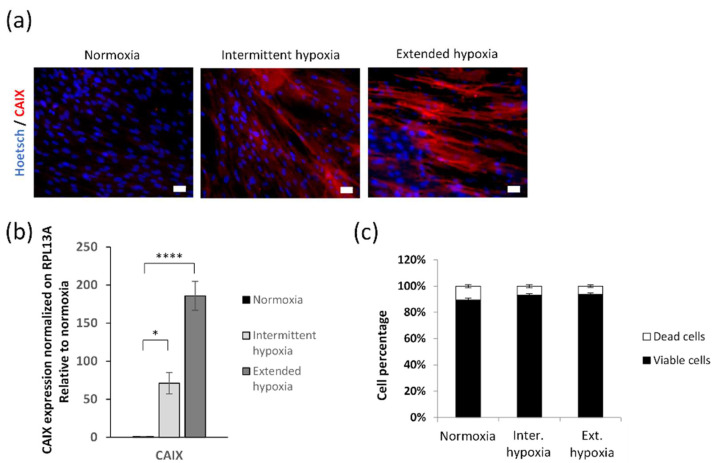
Hypoxia effects on hypoxic marker synthesis and cell viability. (**a**) Immunolabeling of CAIX under normoxia, intermittent and extended hypoxia. Scale bars = 20 µm. (**b**) RT-qPCR of CAIX normalized by RPL13A. Mean ± SEM, *n* = 4 or 6 per group. Statistical tests by ANOVA with Dunnett’s multiple comparisons test, * *p* < 0.05 and **** *p* < 0.0001. (**c**) Viable and dead cell counting with trypan blue.

In order to confirm hypoxic cell conditions, we investigated carbonic anhydrase IX (CAIX), a marker of hypoxia, and cell viability. CAIX expression positively labeled the hypoxic responses of cells, either when grown in intermittent and extended hypoxia conditions (Figure 1a), corresponding to 71- and 185-fold increases, respectively, compared to CAIX mRNA expression level under normoxia (Figure 1b). Hypoxia at 1% pO_2_ under intermittent or extended conditions did not affect cell viability (Figure 1c). 

### 2.2. Hypoxia Decreases Elastic Fibers Deposition

**Figure 2:**
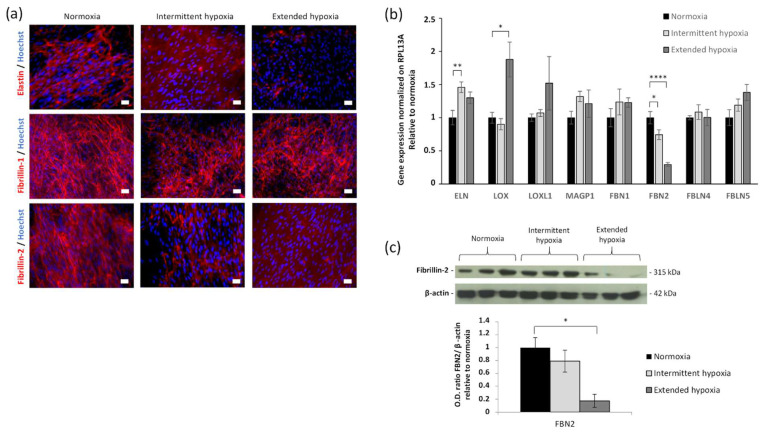
Effect of hypoxia on ELN and FBN2 synthesis and deposition in human primary fibroblasts. (**a**) Immunolabeling of ELN, FBN1 and FBN2 (red, Alexa A546) under normoxia, intermittent and extended hypoxia. Nuclei counterstained with Hoechst. Scale bars = 20 µm. (**b**) RT-qPCR under normoxia, intermittent and extended hypoxia of elastic fiber-related genes normalized by RPL13A and relative to normoxia. Mean ± SEM, *n* = 4 or 6 per group. Statistical tests by ANOVA with Dunnett’s multiple comparisons test for each gene, * *p* < 0.05, ** *p* < 0.01 and **** *p* < 0.0001. (**c**) Western blotting for FBN2 with β-actin as loading control under normoxia, intermittent and extended hypoxia. Optical density (O.D.) was measured and reported as mean ± SEM, *n* = 3 per group. Statistical tests by ANOVA with Dunnett’s multiple comparisons test, * *p* < 0.05.

Under normoxic conditions ELN staining was observed on fibers around the cells, whereas it was abolished under both hypoxic conditions (Figure 2a), while a slight but significant increase of its mRNA expression level was observed with intermittent hypoxia (Figure 2b). Elastin fibrillogenesis impairment appeared as a specific phenomenon since hypoxia did not affect fibronectin and type I collagen deposition (Figure S1a,b).

### 2.3. Hypoxia Decreases FBN2 Gene Expression and Protein Deposition

The fact that the extracellular elastin network is disturbed without strong modification of its gene expression level led us to analyze other partners of the elastic fibers to explain this phenomenon. The most significant variations were observed in FBN2 gene expression level for both hypoxic conditions, with a decrease of about 25% and 70%, respectively, for intermittent and extended hypoxia conditions (Figure 2b). The downregulation of FBN2 mRNA expression level was further demonstrated at the protein level, as FBN2 immunodetection dramatically decreased in cell cultures analyzed by immunofluorescence (Figure 2a) and in fibroblast extracts examined by Western blotting for extended hypoxia (Figure 2c). In contrast, the FBN1 expression level (Figure 2b) and deposition (Figure 2a) were not altered under both hypoxic conditions. There was no significant change under both hypoxic conditions for LOXL1, MAGP1, FBLN4 and FBLN5 (Figure 2b). 

### 2.4. Hypoxia Decreases Mature LOX Protein and Activity

**Figure 3:**
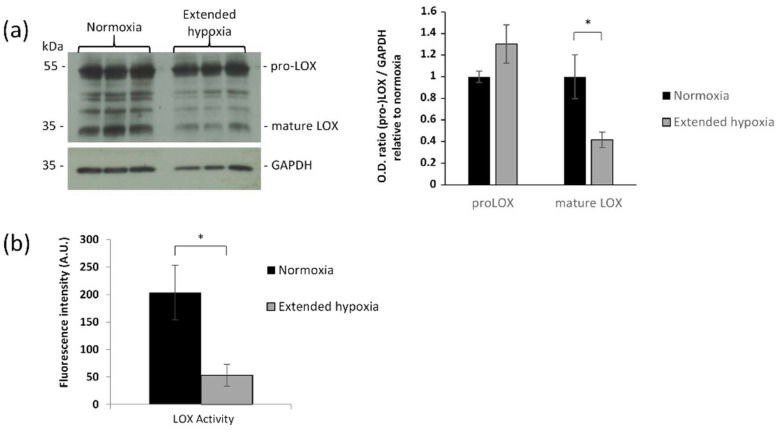
Influence of hypoxia on LOX synthesis, maturation and enzymatic activity in human skin fibroblasts. (**a**) LOX Western blotting with GAPDH used as loading control and semi-quantification. Mean ± SEM, *n* = 3 per group. Statistical tests by multiple unpaired *t* tests, * *p* < 0.05. (**b**) LOX activity determined under normoxia and extended hypoxia (72 h). Mean ± SEM, *n* = 6 per group. Statistical tests by unpaired *t* test with Welch’s correction * *p* < 0.05.

**Figure 4:**
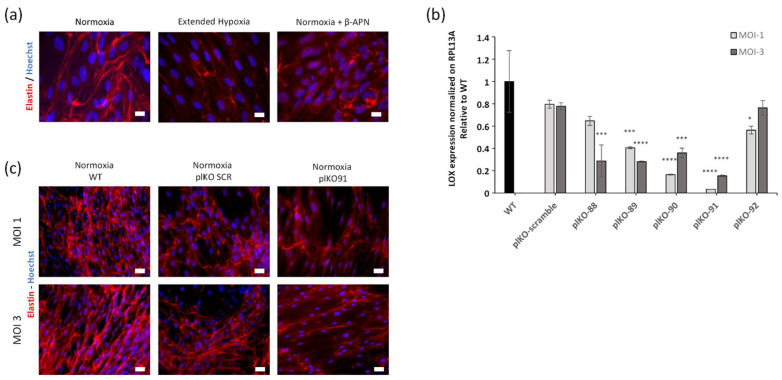
LOX silencing leads to a lack of elastin deposition as in hypoxic conditions with human primary fibroblasts. (**a**) Elastin immunofluorescence from fibroblast under normoxia, extended hypoxia and normoxia + β-APN (350 μM). Nuclei counterstained with Hoechst. Scale bars = 10 µm. (**b**) RT-qPCR of LOX mRNA under normoxia in pLKO shLOX fibroblasts. Mean ± SEM *n* = 3 per group. Statistical test by ANOVA with Dunnett’s multiple comparisons test, * *p* < 0.05, *** *p* < 0.001, **** *p* < 0.0001. Multiplicity of infection (MOI), 1 is one viral particle per cell and 3 is three viral particles per cell. (**c**) ELN immunofluorescence of fibroblasts infected with scrambled (plKO SRC) or sh LOX RNA (plKO-91). Nuclei counterstained with Hoechst. Scale bars = 20 µm.

The steady-state mRNA expression for LOX increased by 1.8-fold in the extended hypoxic condition but not in the intermittent hypoxic condition (Figure 2b). For this reason, the following experiments were conducted in extended hypoxia condition, which also combine the impairments of ELN and FBLN2 depositions. Although LOX expression level was increased, this variation was not associated with a change in pro-LOX (inactive pro-enzyme, 55 kDa) secretion, whereas the mature LOX (active enzyme, 32 kDa) was reduced in extended hypoxic condition (Figure 3a). To confirm the role of LOX activity in the reduction of elastic fibers deposition, we blocked LOX activity using β-aminopropionitrile (β-APN) (Figure 4a) and we also knocked down LOX expression using a short hairpin RNA (shRNA) against LOX under the normoxic condition (Figure 4b). The obtained decrease in LOX activity under normoxia was associated with reduced elastic fiber formation similar to extended hypoxia (Figure 4a). Indeed, among the five different forms of shRNA, the most effective inhibition of LOX by pLKO91 was correlated with a strong reduction in elastic fiber formation in cell culture, similar to that found under extended hypoxia (Figure 4c). Though LOX maturation was reduced, there was no significant expression change under extended hypoxia from its main regulator, BMP-1 (Figure S1c). Therefore, other possible direct effects were considered. Since FBN2 was downregulated under both hypoxic conditions, we thus explored its consequences on LOX maturation.

### 2.5. FBN2 Silencing under Normoxia Recapitulates In Vitro Hypoxia Condition with Both Reductions in Elastic Fiber Deposition and LOX Maturation

**Figure 5:**
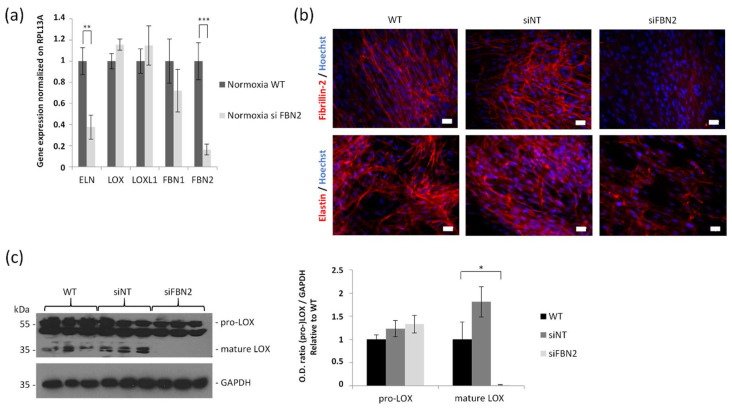
Effect of FBN2 knockdown on elastic fiber synthesis and lysyl oxidase maturation in human skin fibroblasts. (**a**) RT-qPCR of principal genes of interest under normoxia in fibroblasts treated or not with FBN2 siRNA. Mean ± SEM, *n* = 8 per group. Statistical tests by multiple *t* tests, ** *p* < 0.01, *** *p* < 0.001. (**b**) Immunolabeling of FBN2 and ELN in red (Alexa A546). Nuclei counterstained with Hoechst. Cells were untransfected (WT), transfected with non-target siRNA (siNT) or with siRNA against FBN2 (siFBN2). Scale bars = 20 µm. (**c**) LOX Western blotting, GAPDH as loading control. Cells were untransfected (WT), transfected with non-target siRNA (siNT) or with siRNA against FBN2 (siFBN2). Mean ± SEM, *n* = 3 per group. Statistical tests by two-way ANOVA with Dunnett’s multiple comparisons test, * *p* < 0.05.

Fibroblasts were transiently transfected by siRNA blocking FBN2 gene expression. This pool of siRNA inhibited about 90% of FBN2 mRNA expression (Figure 5a). In this analysis, FBN1 was not significantly reduced while a significant decrease in the ELN steady-state mRNA level was observed (about 60%, Figure 5a). Correlating with these results, elastic fibers’ formation was almost completely abolished upon blocking FBN2 gene expression (Figure 5b). Interestingly, LOX protein expression was not significantly modified (Figure 5a), though the complete inhibition of LOX maturation was observed under FBN2 siRNA conditions (Figure 5c).

### 2.6. In Vivo Skin Hypoxia Reduced ELN and FBN2

**Figure 6:**
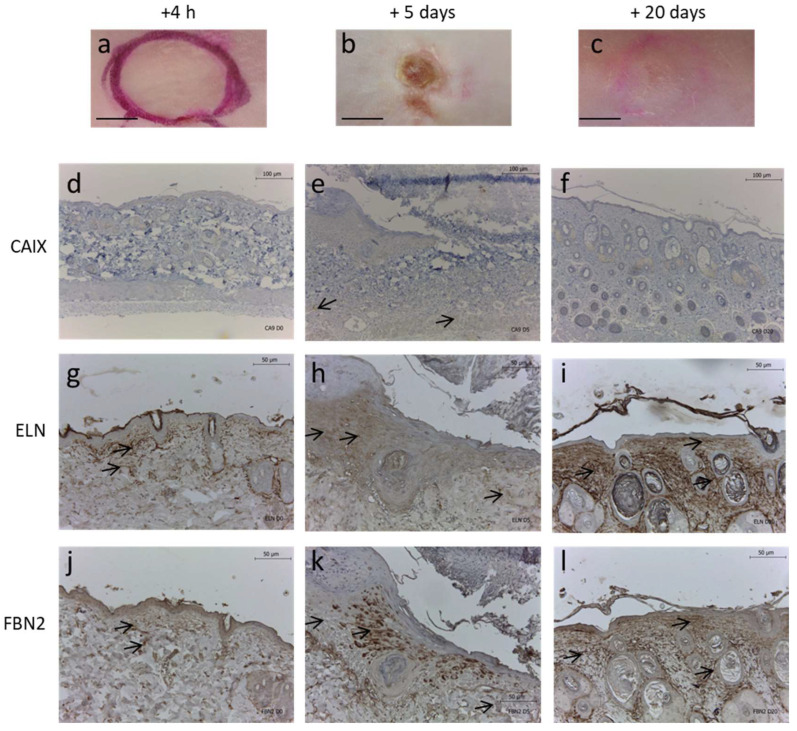
Effect of pressure-induced hypoxic ulcer model on ELN and FBN2 fibers in mice. Representative images of mouse dorsal skin at (**a**) 4 h, (**b**) 5 days and (**c**) 20 days after removal of magnet compression. Corresponding skin immunohistochemistry for (**d**–**f**) CAIX, (**g**–**i**) ELN and (**j**–**l**) FBN2. Scale bars = (**a**–**c**) 4 mm, (**d**–**f**) 100 µm, and (**g**–**l**) 50 µm.

To further explore the relationship between hypoxia and ELN synthesis regulation we performed a pressure-induced skin hypoxic ulcer model in mice. After removal of the magnet (Figure 6a) the wound became necrotic at day 5 (Figure 6b), and at day 20 a scar was hardly visible (Figure 6c). Hypoxic regions using CAIX expression staining were observed at removal of the magnet (Figure 6d); they remained strongly detected in the wound area at day 5 (Figure 6e) and were no longer detected at day 20 (Figure 6f). 

At removal of the magnet, ELN was immunodetected on fiber structures within the connective tissue below the epidermis and around the skin appendages (Figure 6g). The ELN staining was greatly reduced at day 5 (Figure 6h) but reverted to normal levels at day 20 (Figure 6i). These observations suggested an impairment in elastic fiber network at the site of pressure-induced hypoxic ulcer injury, and the neosynthesis of elastic fibers occurred during wound healing and could be detected at day 20. 

At removal of the magnet, FBN2 fibers were detected in the connective tissue under the epidermis (Figure 6j) as seen previously for ELN. Then, FBN2 staining was reduced at day 5 (Figure 6k) and was back at day 20 (Figure 6l).

All the changes observed in ELN and FBN2 immunostaining from skin compression to 20 days are concordant with the kinetic of tissue healing process. In addition, recovery from skin compression was observed 20 days post compression while the metal plate was still in place subcutaneously, excluding any confounding effect.

ELN staining appeared to be correlated with FBN2 staining at wound closure (at day 20) (Figure 6i,l).

## 3. Discussion

This study addresses an important question in wound healing and regenerative medicine regarding the absence or reduction in elastic fiber neo-formation during wound healing and tissue repair. This is a long-standing problem for which no satisfactory solution has been found yet. Few models have been designed to study chronic or long-term ischemia/hypoxia mimicking the conditions within chronic wounds, ulcers or during tissue regeneration. We therefore developed a combined strategy of (i) in vitro models using post-confluent skin fibroblasts under moderate to extended hypoxic conditions, and (ii) an in vivo pressure-induced hypoxic wound in mice, displaying dermal hypoxic sites. The results showed that hypoxia specifically impaired elastin but not collagen network formation. The hypoxia-related elastic fiber defect was closely associated to a reduced FBN2 staining both in vitro and in vivo and confirmed by protein and gene expression reduction in vitro. 

Within normal skin, in vivo percentage of O_2_ is evaluated at around 9% to 10% [21,22]. Therefore, control conditions set at 21% were rather hyperoxic. Based on the principles of biological O_2_ distributions, it is impossible to faithfully reproduce the O_2_ concentration specific to a tissue in an incubator. It is therefore accepted that, for hypoxia studies, it is necessary to compare a “normoxic” condition where no hypoxic cell marker is activated (HIF and its targets such as CAIX for example), with a “hypoxic” condition where these markers are active without affecting cell viability [23]. Thus, it is the state of the cell O_2_ sensing system that guides the experimental conditions. In our model of post-confluent fibroblasts, there was little difference between 21% and 5% of O_2_ regarding elastic fiber deposition (data not shown). However, under 1% hypoxic condition, which is also the lower limit that can be reached in our incubator, there was no further deposition of elastic fibers, even during intermittent periods of normoxia and hypoxia. Interestingly for this study, cell viability was not affected, as shown previously [24]. Therefore, 1% of O_2_ was efficient to specifically decrease elastic fibers since it did not affect type I collagen deposition and network formation.

In the present study, the elastic fibers network was decreased either in human skin fibroblasts under hypoxia, in accordance with results obtained in rat lung fibroblasts [18,25], or in the present mouse hypoxia skin model. Indeed, to further understand of the effect of hypoxia on the elastin network in vivo, a skin pressure ulcer model was developed by adapting the protocol [26,27,28,29]. The mouse skin pressure ulcer model has been shown to display sites of hypoxia that could be observed directly after the end of compression [20], which remains present after 5 days in the present study and was associated with elastin deficit. In Berks’ study [18], a decrease in ELN mRNA expression level was observed after 3 days of culture under hypoxia at a time when the mRNA expression level was maximal under normal conditions. In our model, after 8 days of post-confluence under extended hypoxia, the steady-state ELN mRNA expression level was not statistically reduced. This difference might be explained by the time at which the investigations were conducted, since the time course of ELN expression has been shown to reach a plateau after more than 2 or 3 days after confluence [14]. In any case, the result was the same with no elastic fiber formation under hypoxia, suggesting that indirect mechanisms of hypoxia on ELN gene regulation could be at work. 

Several proteins contribute to connect ELN to the ECM or to cells, such as fibulins, MAGP1 or emilins [30,31]. Fibrillins are also essential as they constitute the microfibril network on which the tropoelastin is deposited for further maturation. This present study highlights an essential role for FBN2, but not FBN1, since FBN2 gene expression was reduced by 70% under extended hypoxia associated with a dramatic immunodetection reduction in vitro. In vivo, at day 5, FBN2 was only observed sparsely in hypoxic tissues marked by the presence of CAIX, a protein used as a specific and stable marker of chronic hypoxia [32].

Therefore, in the present study, the in vitro and in vivo experiments reveal that hypoxia induced a decrease in dermal elastic fibers associated to FBN2 staining reduction. 

To confirm the association between FBN2 and elastin, we silenced FBN2 in fibroblasts and found a dramatic reduction of elastin network under extended hypoxia. Indeed, the in vitro inhibition of FBN2 gene expression by 90% clearly abrogated the ELN mRNA expression. 

Surprisingly, the FBN2 gene expression decreased dramatically although HIF1 was found to regulate four promoters in FBN2 [33] and hypoxia generally increases the transcription of subsets of genes [34]. This discrepancy could reveal an indirect effect of hypoxia on FBN2 decreased expression via other regulators, such as increased miR-9-5p, as reported in kidney cells [35].

FBLN4 and FBLN5, which are accessory proteins in elastogenesis, appear not to be affected by both hypoxic in vitro conditions. The strong binding between FBLN4 and LOX enhanced the interaction of FBLN4 with tropoelastin, forming ternary complexes that may direct elastin cross-linking [36]. Therefore, if we consider the temporal sequence of elastic fiber formation, we can see that proteins involved in the first step, like fibrillins, seem to be more affected than others. 

The formation of elastic fibers is complex and involves several post-translational regulations and protein assembly steps. An interesting finding of this study concerns the regulation of LOX maturation and activity. First, LOX mRNA expression appeared to be stimulated by hypoxia. LOX has been identified as one of the best markers of hypoxia for several epithelial cells [19]. However, in such a situation, LOX has been described as pro-tumorigenic and pro-metastatic through its mature and active region, while the pro-region acts inversely as an anti-tumor factor [19,37]. The situation appears quite different for mesenchymal cells, where LOX gene expression appears to be only slightly affected by hypoxia [19]. Here, we found that LOX expression and maturation are essential for the formation of elastic fibers, using our in vitro cell hypoxic model and shRNA against LOX that cannot be compensated by LOXL1. The exact role of each enzyme of the LOX family is not yet well defined. However, LOX and LOXL1 have both been closely associated with the formation of elastic fibers [38,39]. LOX activity is important for the cross-linking of many collagens, though irregular collagen fibers can be formed and deposited when it is inhibited [40,41]. It has been generally accepted from in vitro studies that BMP-1 or related enzymes are processing the pre-proenzyme to produce an active mature LOX [42,43]. In our in vitro cell extended hypoxia model, the BMP-1 protein level was not changed by hypoxia (Figure S1c) and thus appeared not to be involved in the decrease of mature LOX. In addition, while direct interactions between LOX and tropoelastin, and ELN and FBN1 have been shown [14,44], interactions between LOX and FBN2 have never been documented and pave the way of a new molecular mechanism for LOX activity regulation. 

The mouse model allowed us to observe the neosynthesis of elastic fibers with progressive reversal of tissue hypoxia, a situation rarely observed in wound healing. In vivo at day 20 post-compression, the CAIX hypoxia staining marker was largely reduced in contrast to both ELN and FBN2 staining. In addition, we never found situations where ELN staining was strong and FBN2 staining was low. Thus, the in vivo studies were in accordance with the in vitro studies regarding FBN2 and ELN changes, depending on hypoxia levels and confirmed the close relationship between those two proteins. Berck et al. [25] reported that hypoxia suppressed elastin repair at all time points of recovery following protease exposure. The present animal setting confirms that tissue hypoxia removal allows for elastin repair and highlights the key role of FBN2 in this mechanism. In vivo, FBN2 has been shown to be located at the edge of acute wounds, concomitant with an increase in TGF-β, suggesting a role during healing [45]. Numerous studies have shown that in wound healing, there is a phenomenon of reprogramming that leads to embryonic protein synthesis such as collagen types I, III and V [46]. FBN2, which is involved in the first steps of microfibrils formation, has been specifically described as a protein expressed during development and the first period of growth, whereas FBN1 is expressed throughout life. Indeed, in the context of fetal wound healing, elastic fiber network is usually produced [8]. Brinckmann et al., (2010) showed a re-induction of FBN2 and tenascin-C synthesis during acute wound healing and in cases of sclerosis. Interestingly, these two proteins are predominantly synthesized and involved in development [47,48]. The same observation was made in a study that reported the production of tenascin-C in wound healing [49].

## 4. Materials and Methods

### 4.1. Cell Culture

For this study, commercial normal human dermal fibroblasts derived from healthy neonatal donor foreskin were used (PCS-201-010 from ATCC, LGC Standards, Molsheim, France).

Primary human dermal fibroblasts were cultured in a mixture of Dulbecco’s Modified Eagle’s Medium/Ham’s F12 nutrient mixture (DMEM/F12 1:1) supplemented with 10% fetal bovine serum (FBS) and penicillin-streptomycin (Life Technologies, St Aubin, France). Cells were incubated at 37 °C under normoxia (5% CO_2_, 21% O_2_ and 74% N_2_) or hypoxia (5% CO_2_, 1% O_2_ and 94 % N_2_) using a dual-gas (CO_2_/N_2_) incubator (Binder CB-150) including CO_2_ and O_2_ probes. Two protocols were designed as follows: (1) four cycles of a phase of one day of normoxia followed by hypoxia the next days, called “intermittent hypoxia”; and (2) a prolonged phase of hypoxia during 8 days, called “extended hypoxia”.

A cDNA-encoding human LOX (Genescript Biotech, Leiden, The Netherlands) was cloned into the retroviral vector plasmid pSLIK (ATCC, LGC Standards, Molsheim, France). Primary dermal fibroblasts were infected with Mission RNAi lentiviral shRNA particles targeting LOX (TRCN0000045991, shLOX) and non-targeting control sequences (SHC002V, shCtl) purchased from Sigma-Aldrich (Saint-Quentin-Fallavier, France). All infections were carried out at low multiplicity of infection (MOI) in a medium with 8 µg/mL polybrene. Infected cells were selected with 1 µg/mL puromycin (Life Technologies, St Aubin, France). 

Primary dermal fibroblasts transfected with FBN2 siRNA (L-011656-00-0005) and the non-targeting control (D-001810-10-05) were cultured under normoxia as recommended by the manufacturer (Thermo Fisher Scientific, Villebon sur Yvette, France).

### 4.2. Viability Test

Fibroblasts were centrifuged and suspended in 1X phosphate-buffered saline (PBS). Dead cells were stained with Trypan blue and counted using a hemocytometer.

### 4.3. Immunofluorescence

Cells were fixed and permeabilized with 10% methanol at −20 °C for 20 min. After saturation with 5% BSA in PBS, cells were stained for CAIX (NB100-417 Novus Biological, Cambridge, UK), elastin (Ab21607Abcam, Cambridge, UK), collagen I (Novotec, Bron, France), fibrillin-2 (a mouse anti-human fibrillin-2 polyclonal antiserum) and fibrillin-1 (MA1-21805 Thermo Fisher Scientific, Villebon sur Yvette, France) for DNA (Hoechst, Sigma-Aldrich, Saint Quentin Fallavier, France) and Alexa 546 as a secondary antibody (A-21089, Thermo Fisher Scientific, Villebon sur Yvette, France). 

### 4.4. Western Blotting

Fibroblasts were lysed with 8 M urea/10% NP-40/0.2 M EDTA/protease inhibitor cocktail/0.5 M Na_2_HPO_4_ pH 7.5. Lysates were collected and pelleted by centrifugation (30,000× *g*). Salts and proteins were precipitated with 10% trichloroacetic acid, centrifuged, rinsed with acetone, pelleted, dried and suspended in RIPA buffer (50 mM Tris-HCl, pH 8.0, 150 mM NaCl, 1% NP-40, 0.5% sodium deoxycholate, 0.1% SDS, 1 mM Na_3_VO_4_, protease cocktail inhibitor (Sigma-Aldrich, Saint Quentin Fallavier, France)). Protein concentrations were measured using the Pierce BCA method, as recommended by the manufacturer. Proteins were then loaded on polyacrylamide gels in Laemmli sample buffer. Membranes were blocked with 5% non-fatty milk after transfer and incubated with primary antibody FBN2 (HPA012853, Sigma-Aldrich, St Quentin Fallavier, France) and LOX [38], washed with 1X tris-buffered saline (TBS) with 0.1% Tween and incubated with a secondary antibody coupled to horseradish peroxidase (HRP) (BioRad, Marnes-la-coquette, France). The signal was revealed by enhanced chemiluminescence (Thermo Fisher Scientific, Villebon sur Yvette, France).

### 4.5. Real-Time RT-PCR

Total RNA was purified using Tri-Reagent (Euromedex, Souffelweyersheim, France) according to the manufacturer’s instructions and quantified using a Nanodrop spectrophotometer (Thermo Fisher Scientific, Villebon sur Yvette, France).

Then, 1 µg of RNA was reverse transcribed with a Fermentas kit (K1622, Euromedex, Souffelweyersheim, France) and quantitative real-time PCR was performed. The fragments were amplified using the two-step RotorGene program (Qiagen, Les Ulis, France): 5 min at 95 °C, 50 cycles of 10 s at 60 °C and 30 s at 95 °C, with the measurement of SYBR Green fluorescence at the end of each cycle; then, 1 min at 60 °C, 1 min at 95 °C and finally with a temperature gradient from 55 °C to 95 °C to determine the melting curve. Gene expression levels were determined using the “delta-delta Ct” method and normalized to levels of RPL13A or GAPDH housekeeping genes.

### 4.6. LOX Activity

LOX activity assay was performed using the AmplexRed kit (A22188, Thermo Fisher Scientific, Villebon sur Yvette, France), following the protocol described by Palamakumbura [50]. Results are given in fluorescence intensity (FI) corresponding to the production of hydrogen peroxide during the enzyme reaction (total amine oxidase).

### 4.7. Skin Compression

Male Swiss mice (*n* = 9, aged 10 weeks) were purchased from Janvier^®^ (Le Genest-Saint-Isle, France). The skin injury was induced by applying and removing a magnet to a dorsal region of mouse skin under which a metal plate was implanted [26]. For this purpose, one week prior to skin compression, each mouse was anaesthetized with 2% isoflurane (Baxter SAS, Maurepas, France) and a transverse incision was made through the full thickness of the dorsal skin, and a disinfected metal implant (300 mm^2^, Guillen, France) was inserted between the skin and the underlying skeletal muscle so there would be no direct contact of the metal plate with the dermis, excluding any effect. The metal plate was positioned so that the incision remained outside the compression zone, and then the skin was closed [20]. Two days prior to skin compression, each mouse was anaesthetized with 2% isoflurane (Baxter SAS, Maurepas, France) and depilated with a cream (Veet, France) to avoid any confounding effects. After a 48 h recovery period, the animals were subjected to skin compression using a magnet applied twice daily (160 mmHg, 2 h each time) with 2 h of reperfusion in between while the mice were free to move in their individual cages [51]. Ink dots were applied around the magnet in order to locate the compressed area after the magnet was removed. Skin biopsy was performed at the end of pressure application and at day 5 and 20 after pressure application (*n* = 3 per timepoint). 

### 4.8. Histology and Immuno-Histology

Mice were euthanized via thiopental overdose, and skin tissue was dissected, fixed overnight in buffered 4% paraformaldehyde solution and embedded in paraffin. Sections of 7 μm were labeled with hematoxylin phloxin safranin, anti-ELN (Novotec 25051, Bron, France), anti-CAIX (NB100-417 Novus Biological, Cambridge, UK) and anti-FBN2 (a rabbit anti-mouse FBN2 polyclonal antiserum) antibodies. Mayer hematoxylin was used as a nuclear counterstain.

### 4.9. Statistical Analysis

Statistical analyses were conducted with Graphpad Prism software. In all analyses *n* comprised between 3 and 8 per group as indicated in the corresponding figure legends. Different parametric statistical tests were applied depending on the data to be analyzed. Comparisons of two groups were made by single or multiple unpaired *t* tests, while ANOVA with Dunnett’s multiple comparisons post hoc tests were performed when more than two groups were compared. Significance was indicated as follows: * *p* < 0.05; ** *p* < 0.01; *** *p* < 0.001; **** *p* < 0.0001.

## 5. Conclusions

The present study demonstrated for the first time the involvement of FBN2 in regulating elastin deposition in adult skin models, and described the specific impact of hypoxia on the elastin network without consequences on collagen and fibronectin networks. We also report that FBN2 silencing dramatically affected LOX maturation that could affect elastin cross-linking and contribute to elastin deposition deficit along with elastin decreased gene expression. We thus revealed the key role of FBN2 under hypoxia and wound healing process through indirect FBN2 regulation by hypoxia that needs more studies, although FBN2 is a developmentally expressed gene and is not predicted to participate to adult tissue regulation. The specific link between hypoxia, FBN2, ELN and LOX in understanding the synthesis and assembly of elastic fibers remains to be determined. Further mechanistic correlations in pathophysiological conditions such as human chronic wounds would provide new therapeutic targets.

## Data Availability

The datasets generated and analyzed during the current study are available from the corresponding authors on reasonable request. The data are not publicly available due to confidential statement with Urgo group.

## Supplementary Materials

The following supporting information can be downloaded at: https://www.mdpi.com/article/10.3390/ijms23031824/s1.
