# Supplementary figures and images for: FBN2 Silencing Recapitulates Hypoxic Conditions and Induces Elastic Fiber Impairment in Human Dermal Fibroblasts
Source: Int J Mol Sci. 2022 Feb 5;23(3):1824. doi: 10.3390/ijms23031824 (PMC8836539; doi:10.3390/ijms23031824)

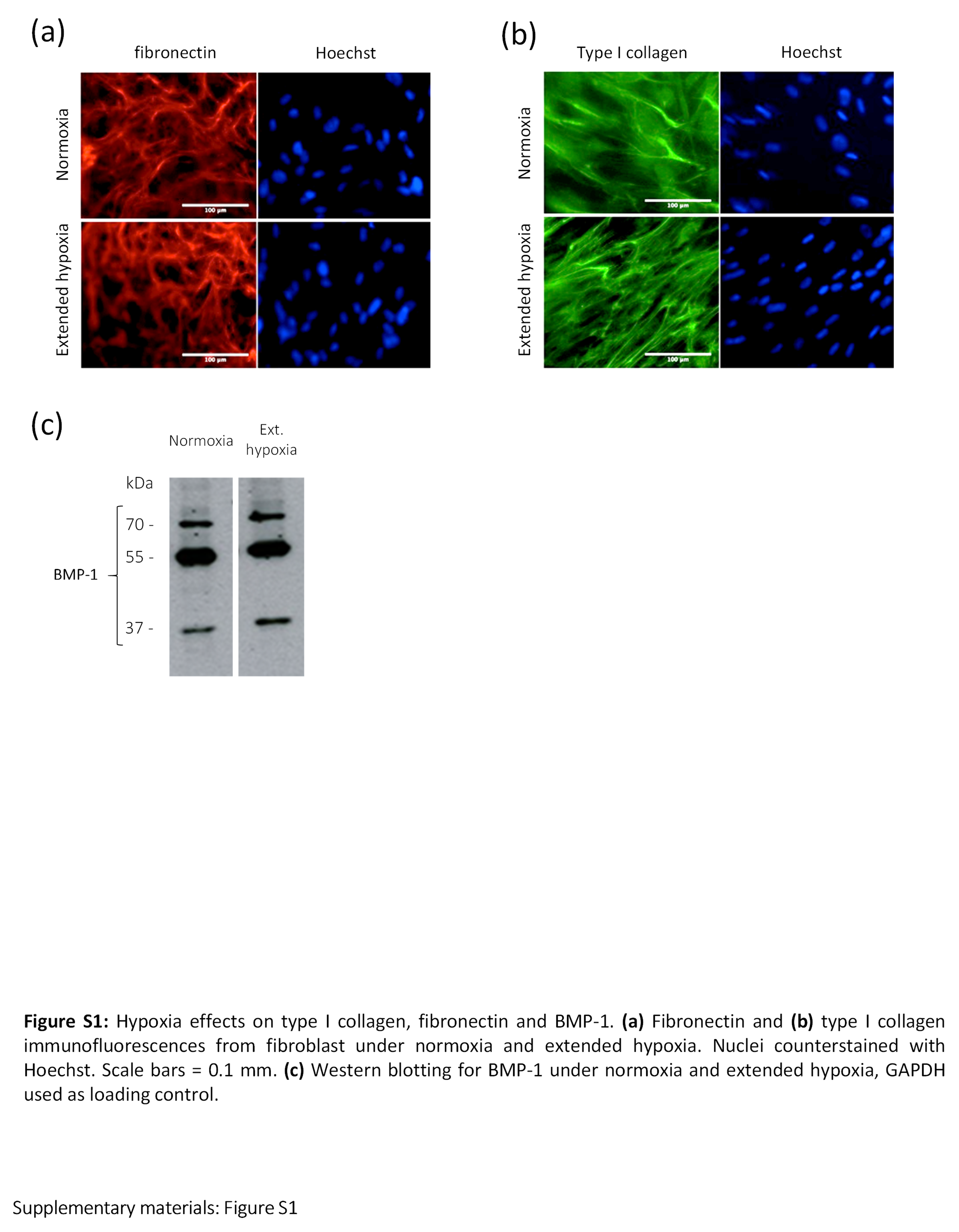

Supplement: Supplementary file 1 [file ijms-23-01824-s001.zip › ijms-1557899-supplementary.tif]
